# Supplementary material for: Effects of Crotonylation on Reprogramming of Cashmere Goat Somatic Cells with Different Differentiation Degrees
Source: Animals (Basel). 2022 Oct 19;12(20):2848. doi: 10.3390/ani12202848 (PMC9597727; doi:10.3390/ani12202848)
Supplement: Supplementary file 1 [file animals-12-02848-s001.zip › Original Images for Blots.pptx]

## Slide 1
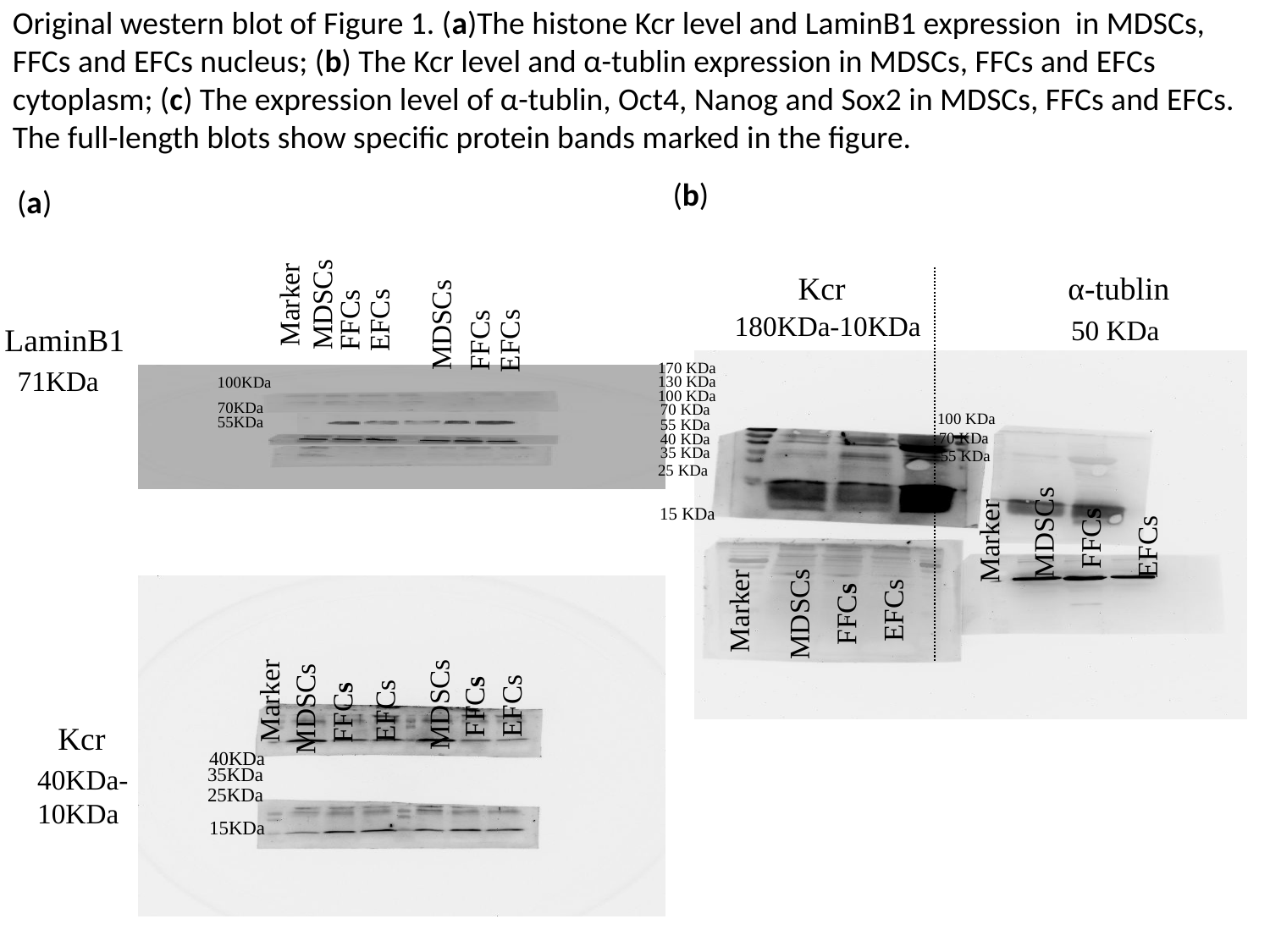

Original western blot of Figure 1. (a)The histone Kcr level and LaminB1 expression in MDSCs, FFCs and EFCs nucleus; (b) The Kcr level and α-tublin expression in MDSCs, FFCs and EFCs cytoplasm; (c) The expression level of α-tublin, Oct4, Nanog and Sox2 in MDSCs, FFCs and EFCs. The full-length blots show specific protein bands marked in the figure.
(b)
(a)
α-tublin
Kcr
Marker
MDSCs
FFCs
EFCs
180KDa-10KDa
MDSCs
50 KDa
LaminB1
FFCs
EFCs
170 KDa
71KDa
130 KDa
100KDa
100 KDa
70KDa
70 KDa
100 KDa
55KDa
55 KDa
70 KDa
40 KDa
35 KDa
55 KDa
25 KDa
15 KDa
MDSCs
FFCs
Marker
EFCs
EFCs
Marker
MDSCs
FFCs
Marker
MDSCs
EFCs
FFCs
MDSCs
EFCs
FFCs
Kcr
40KDa
40KDa-10KDa
35KDa
25KDa
15KDa

## Slide 2
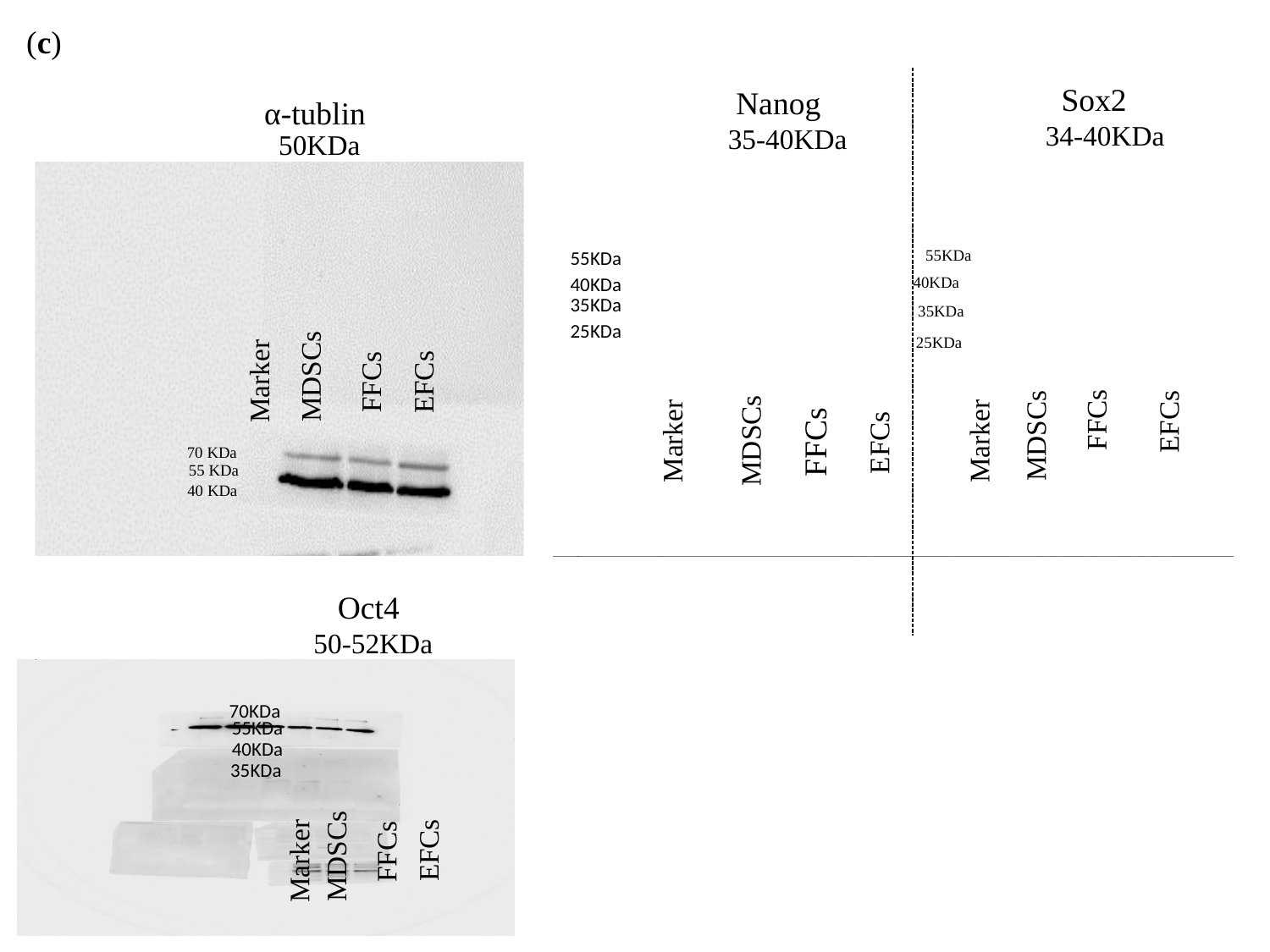

(c)
 Sox2
34-40KDa
 Nanog
35-40KDa
α-tublin
50KDa
55KDa
55KDa
40KDa
40KDa
35KDa
35KDa
25KDa
25KDa
MDSCs
Marker
FFCs
EFCs
FFCs
EFCs
MDSCs
FFCs
MDSCs
Marker
Marker
EFCs
70 KDa
55 KDa
40 KDa
 Oct4
50-52KDa
70KDa
55KDa
40KDa
35KDa
EFCs
FFCs
MDSCs
Marker

## Slide 3
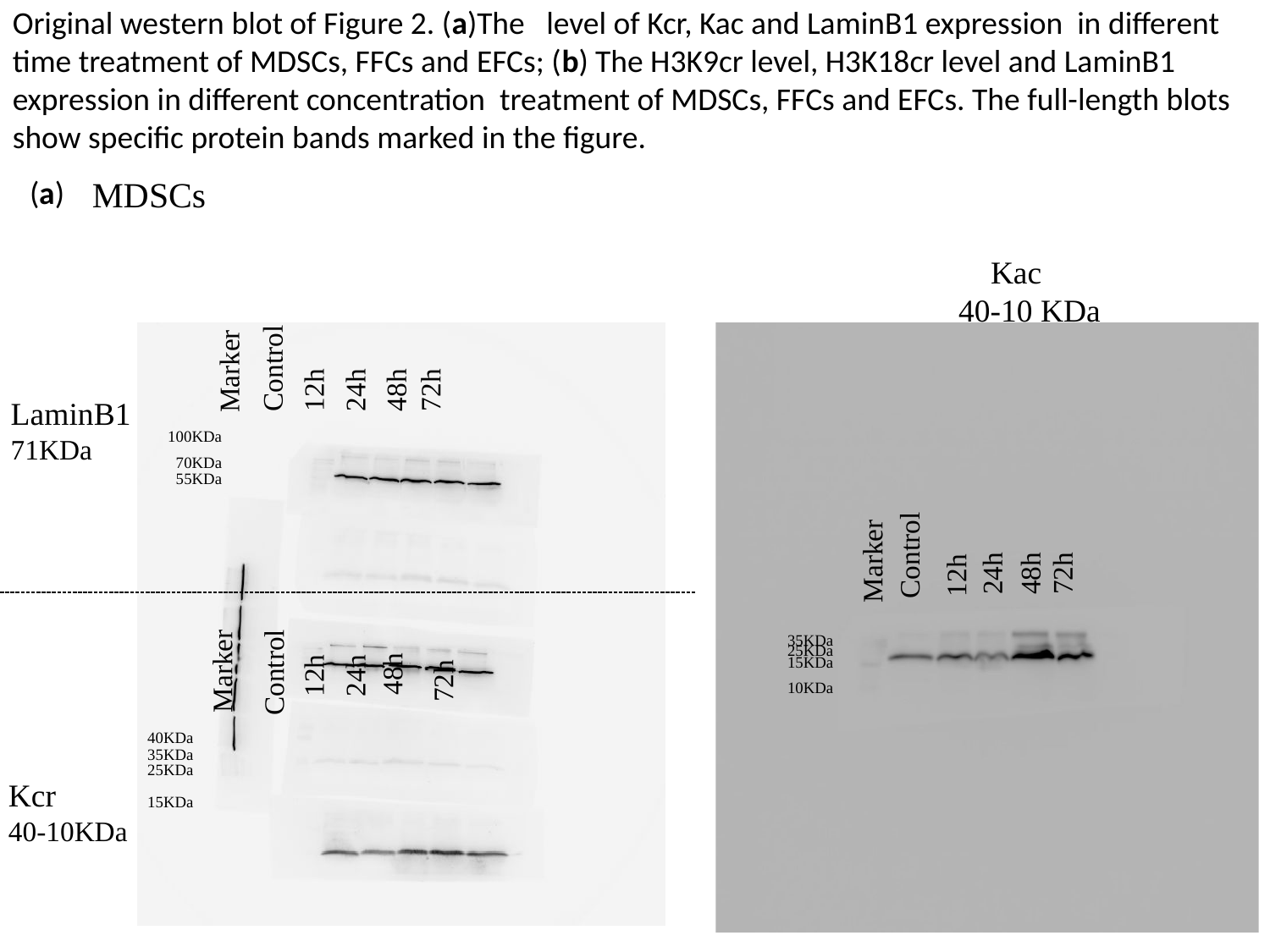

Original western blot of Figure 2. (a)The level of Kcr, Kac and LaminB1 expression in different time treatment of MDSCs, FFCs and EFCs; (b) The H3K9cr level, H3K18cr level and LaminB1 expression in different concentration treatment of MDSCs, FFCs and EFCs. The full-length blots show specific protein bands marked in the figure.
(a)
MDSCs
 Kac
40-10 KDa
Control
Marker
48h
24h
12h
72h
LaminB1
71KDa
100KDa
70KDa
55KDa
Control
Marker
72h
48h
24h
12h
35KDa
25KDa
15KDa
Marker
Control
48h
12h
24h
72h
10KDa
40KDa
35KDa
25KDa
Kcr
40-10KDa
15KDa

## Slide 4
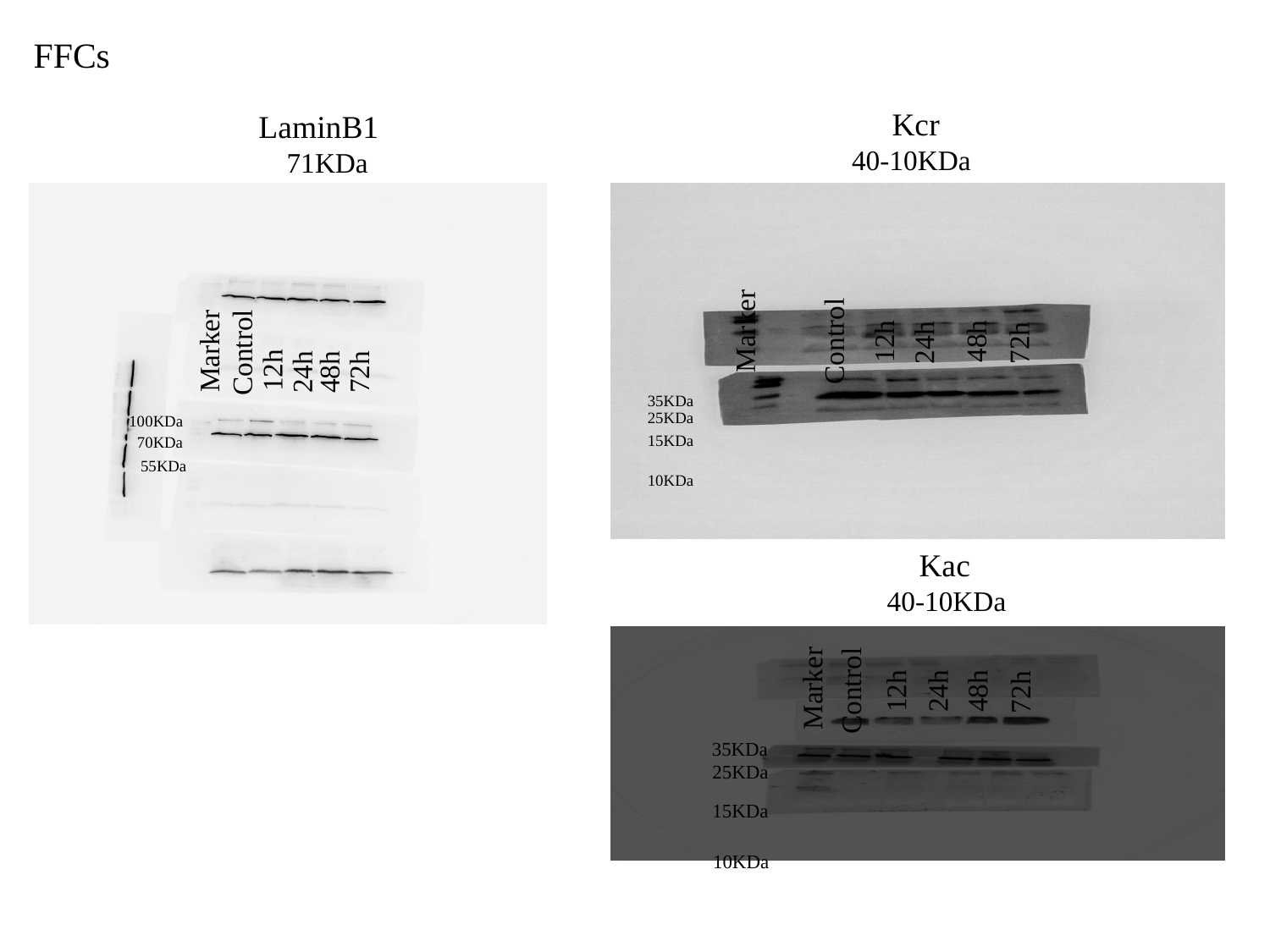

FFCs
 Kcr
40-10KDa
LaminB1
 71KDa
Marker
Control
12h
48h
24h
72h
Marker
Control
12h
24h
48h
72h
35KDa
25KDa
100KDa
15KDa
70KDa
55KDa
10KDa
 Kac
40-10KDa
Marker
24h
12h
Control
48h
72h
35KDa
25KDa
15KDa
10KDa

## Slide 5
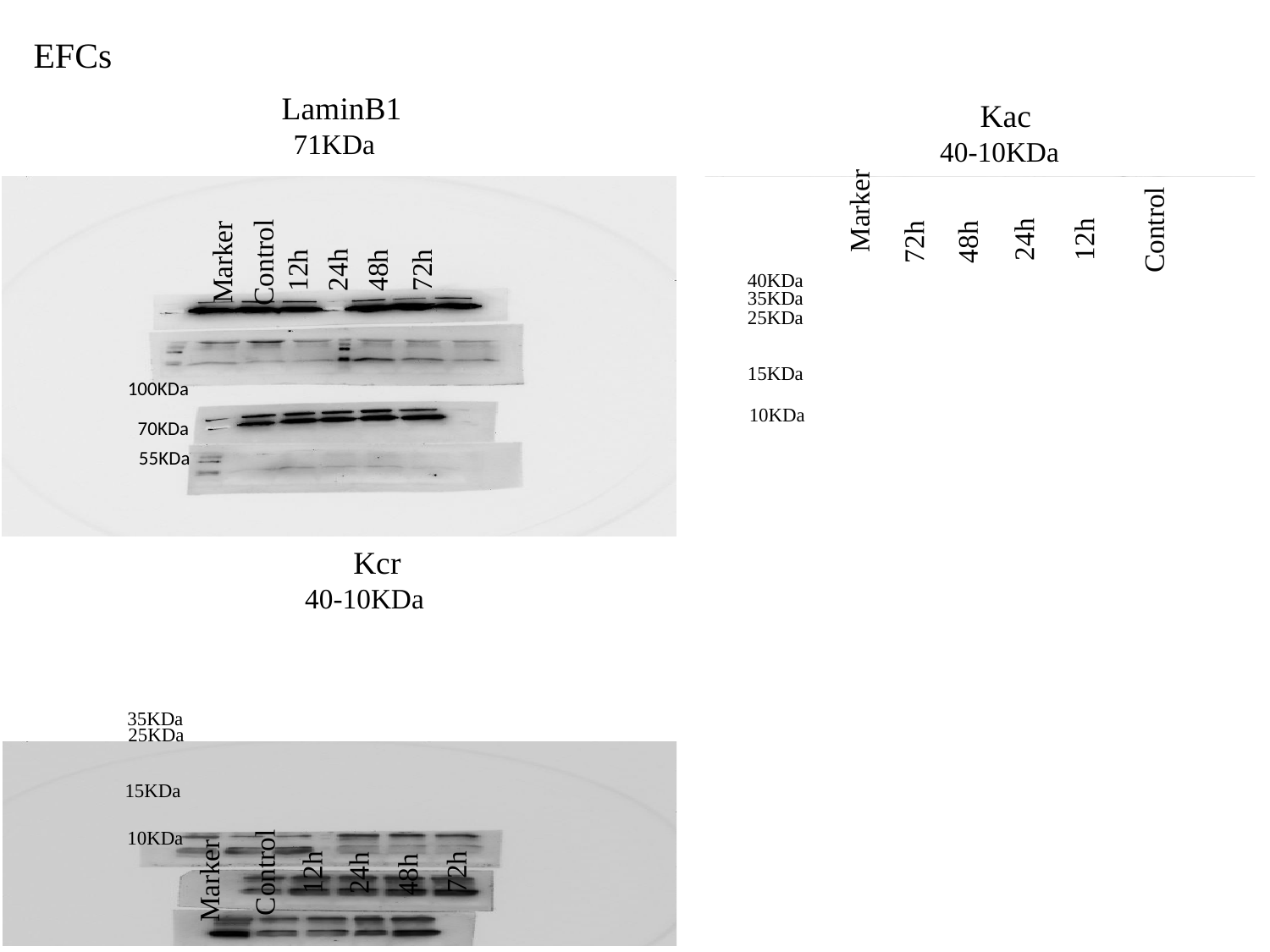

EFCs
 LaminB1
 71KDa
 Kac
40-10KDa
Marker
Control
24h
12h
72h
48h
Marker
Control
12h
24h
48h
72h
40KDa
35KDa
25KDa
15KDa
100KDa
10KDa
70KDa
55KDa
 Kcr
40-10KDa
35KDa
25KDa
15KDa
10KDa
72h
12h
Control
24h
48h
Marker

## Slide 6
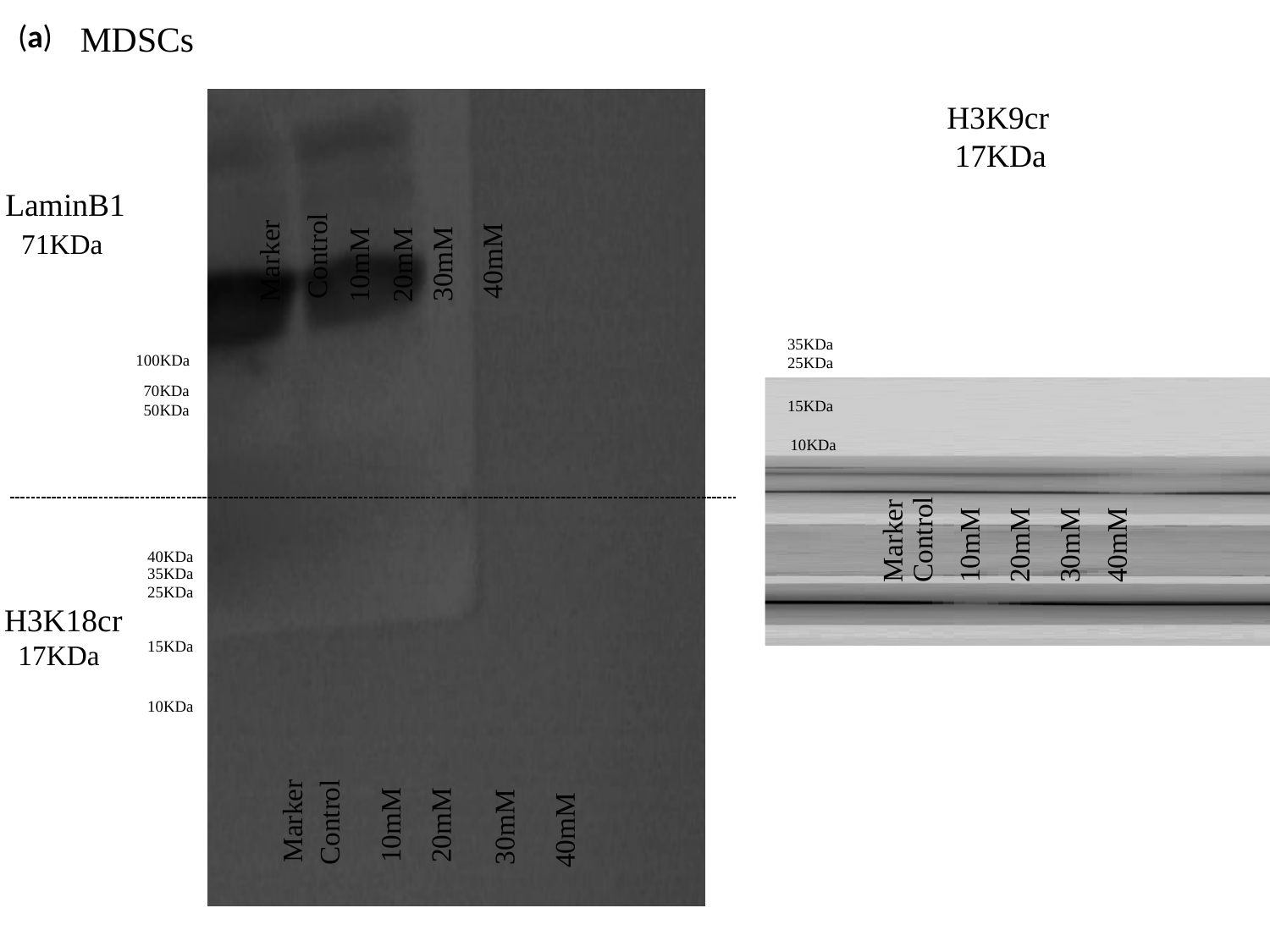

(a)
MDSCs
H3K9cr
 17KDa
LaminB1
 71KDa
Control
40mM
Marker
30mM
10mM
20mM
35KDa
100KDa
25KDa
70KDa
15KDa
50KDa
10KDa
Control
Marker
10mM
20mM
30mM
40mM
40KDa
35KDa
25KDa
H3K18cr
 17KDa
15KDa
10KDa
Marker
Control
10mM
20mM
30mM
40mM

## Slide 7
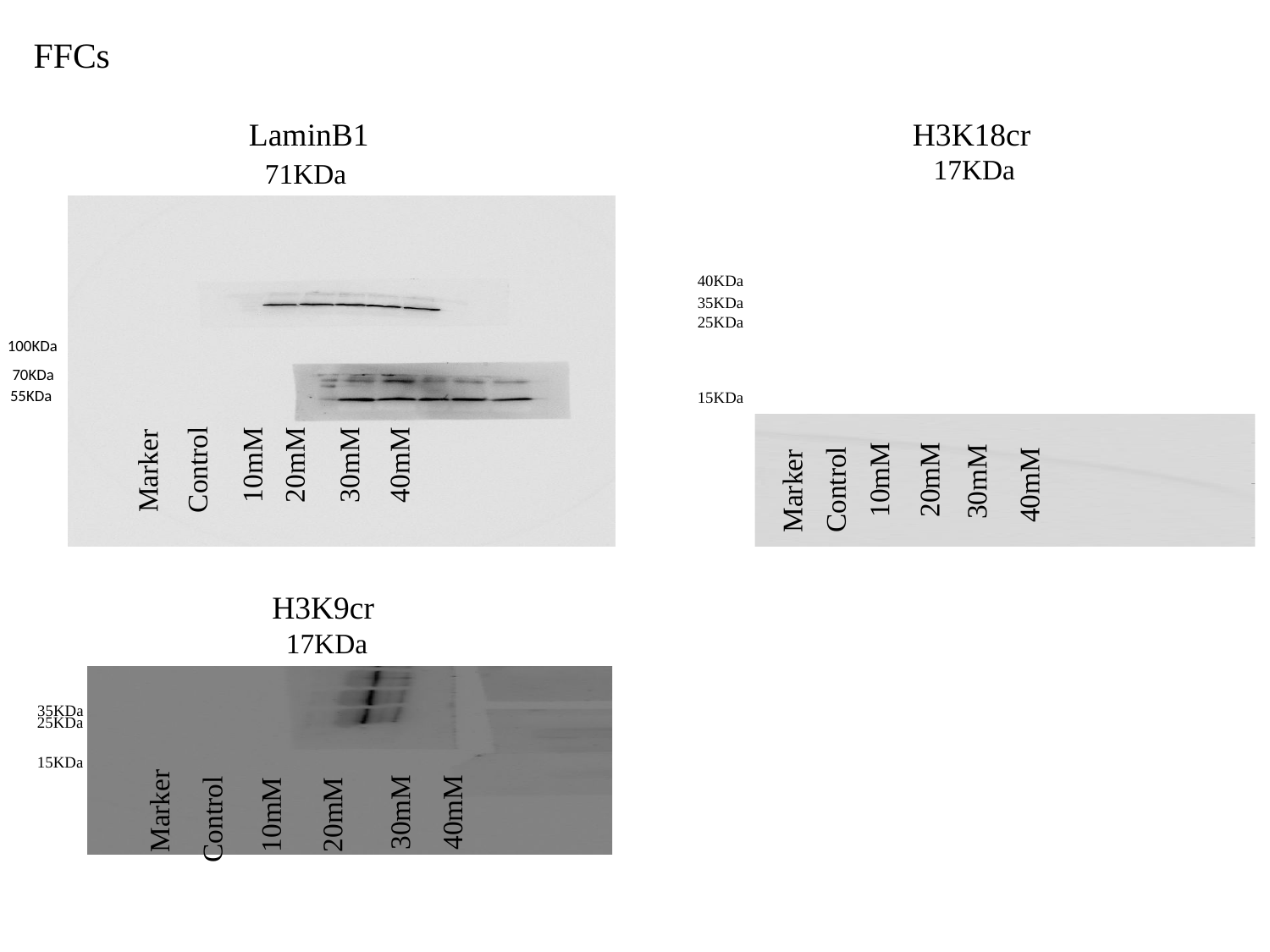

FFCs
LaminB1
 71KDa
H3K18cr
 17KDa
40KDa
35KDa
25KDa
100KDa
70KDa
55KDa
15KDa
10mM
20mM
30mM
40mM
Control
Marker
10mM
20mM
30mM
40mM
Control
Marker
H3K9cr
 17KDa
35KDa
25KDa
15KDa
Marker
30mM
40mM
10mM
20mM
Control

## Slide 8
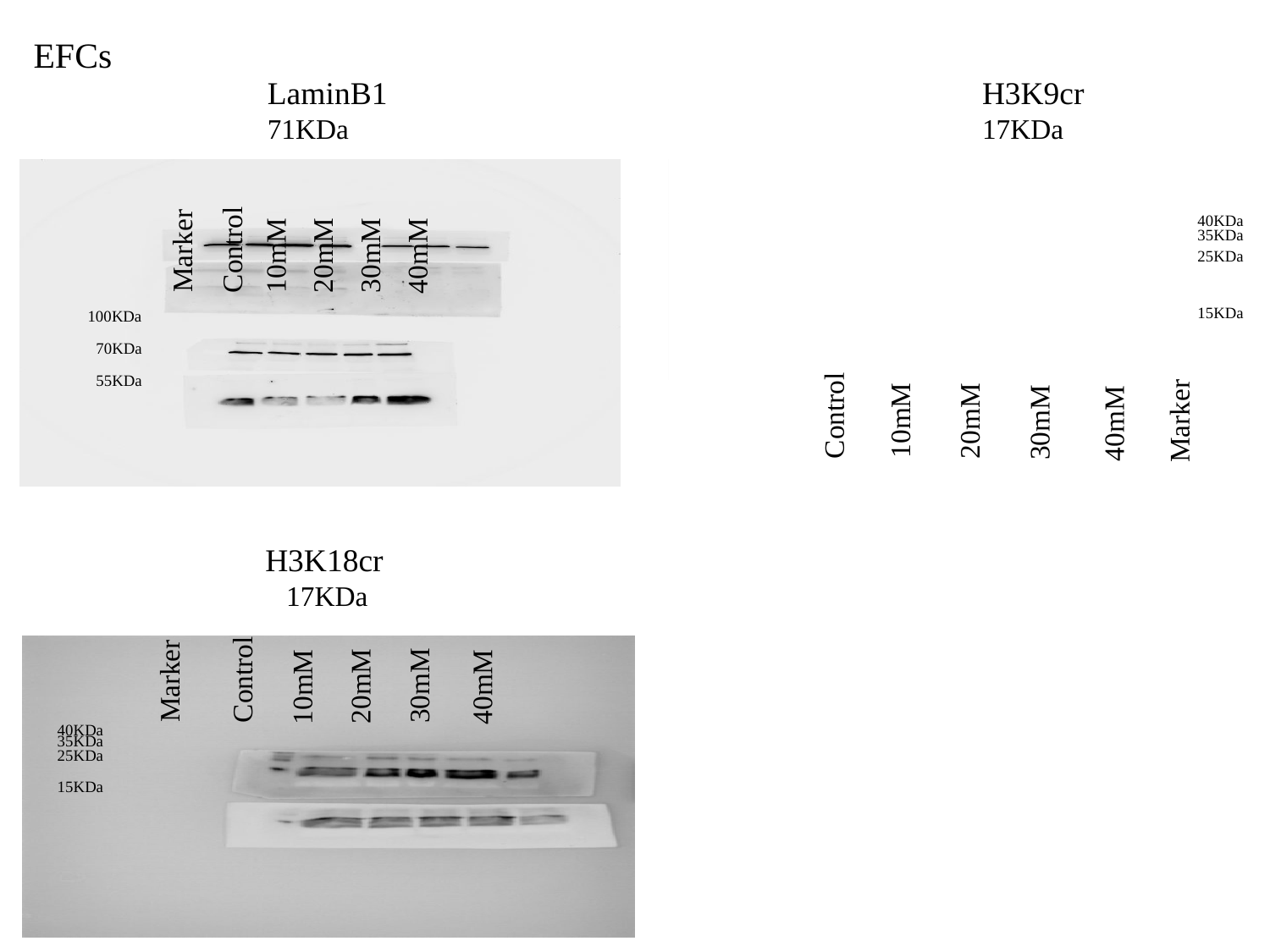

EFCs
LaminB1 71KDa
H3K9cr
17KDa
40KDa
35KDa
Control
Marker
10mM
20mM
30mM
40mM
25KDa
15KDa
100KDa
70KDa
55KDa
Control
10mM
20mM
Marker
30mM
40mM
H3K18cr
 17KDa
Control
Marker
30mM
20mM
40mM
10mM
40KDa
35KDa
25KDa
15KDa
