# Supplementary material for: Effects of Crotonylation on Reprogramming of Cashmere Goat Somatic Cells with Different Differentiation Degrees
Source: Animals (Basel). 2022 Oct 19;12(20):2848. doi: 10.3390/ani12202848 (PMC9597727; doi:10.3390/ani12202848)

## Slide 1
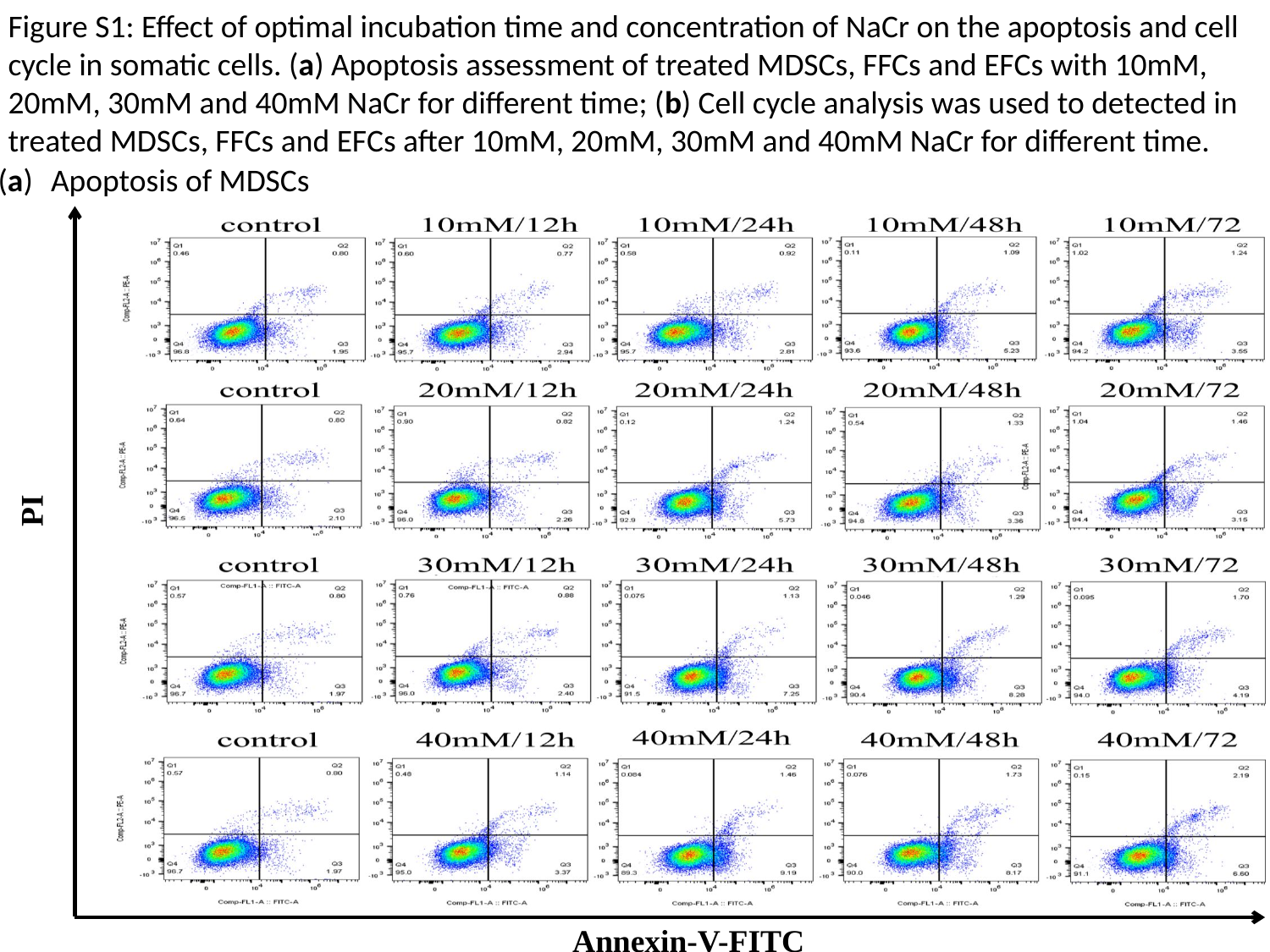

Figure S1: Effect of optimal incubation time and concentration of NaCr on the apoptosis and cell cycle in somatic cells. (a) Apoptosis assessment of treated MDSCs, FFCs and EFCs with 10mM, 20mM, 30mM and 40mM NaCr for different time; (b) Cell cycle analysis was used to detected in treated MDSCs, FFCs and EFCs after 10mM, 20mM, 30mM and 40mM NaCr for different time.
(a)
Apoptosis of MDSCs
PI
Annexin-V-FITC

## Slide 2
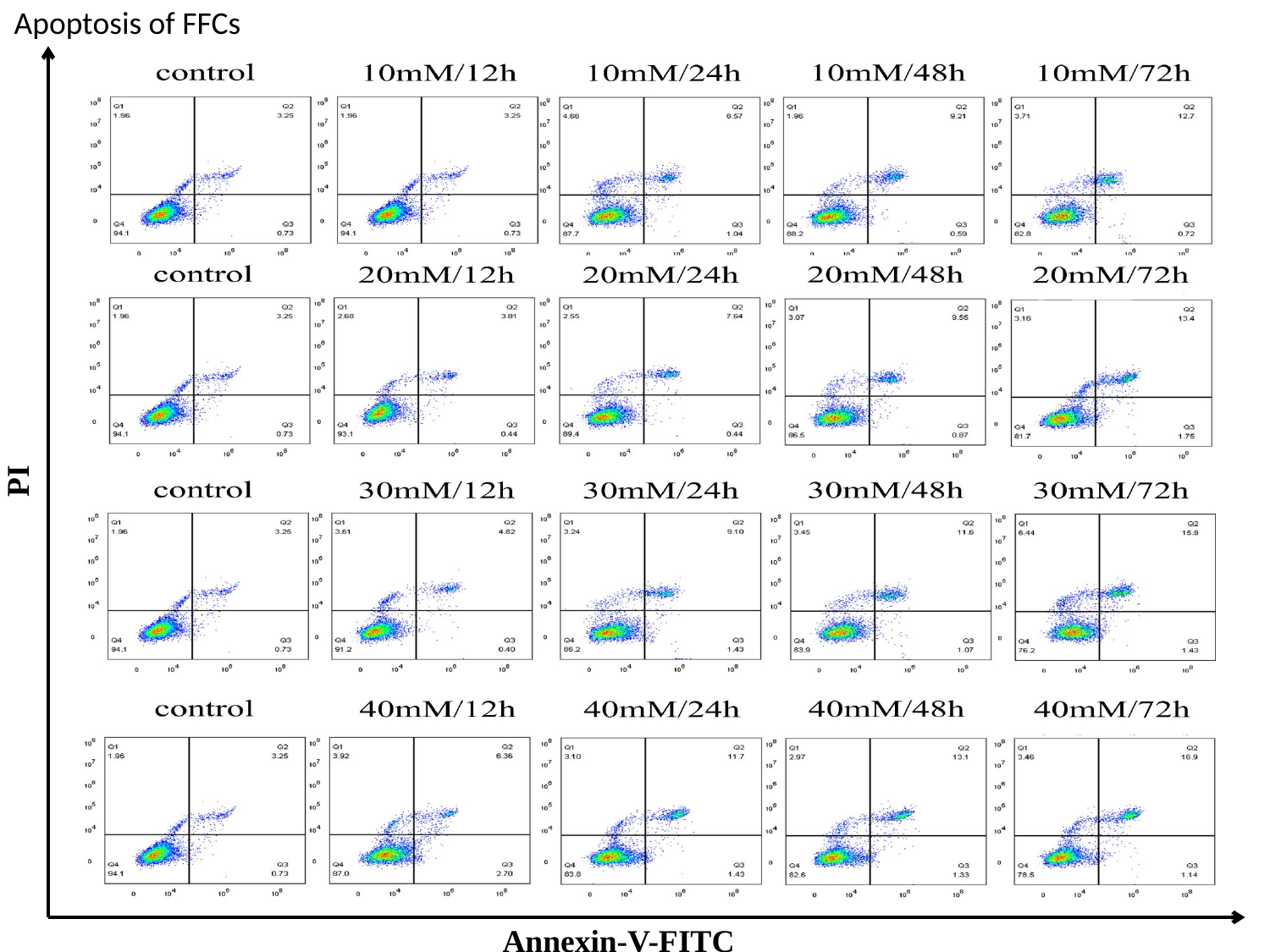

Apoptosis of FFCs
PI
Annexin-V-FITC

## Slide 3
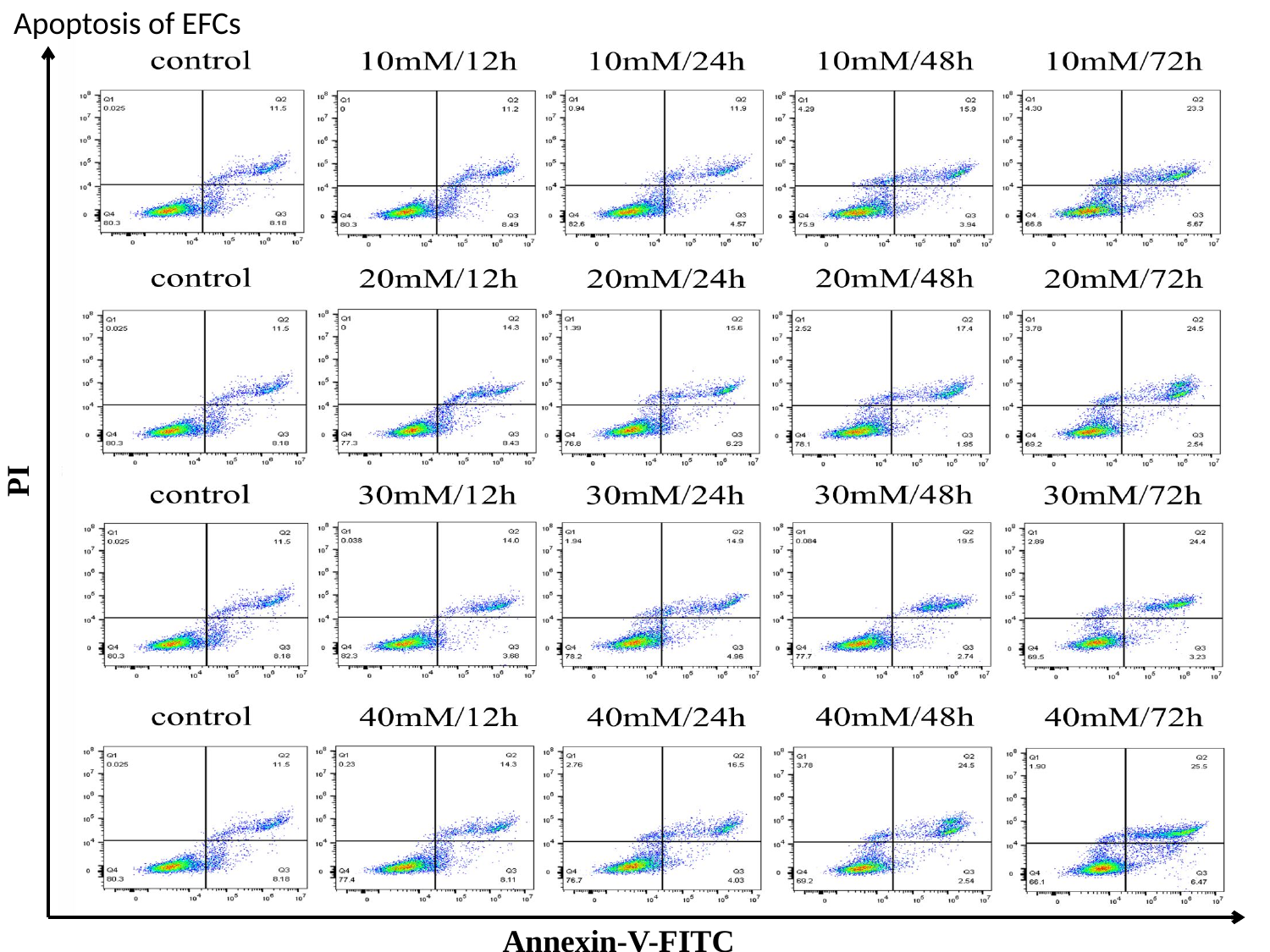

Apoptosis of EFCs
PI
Annexin-V-FITC

## Slide 4
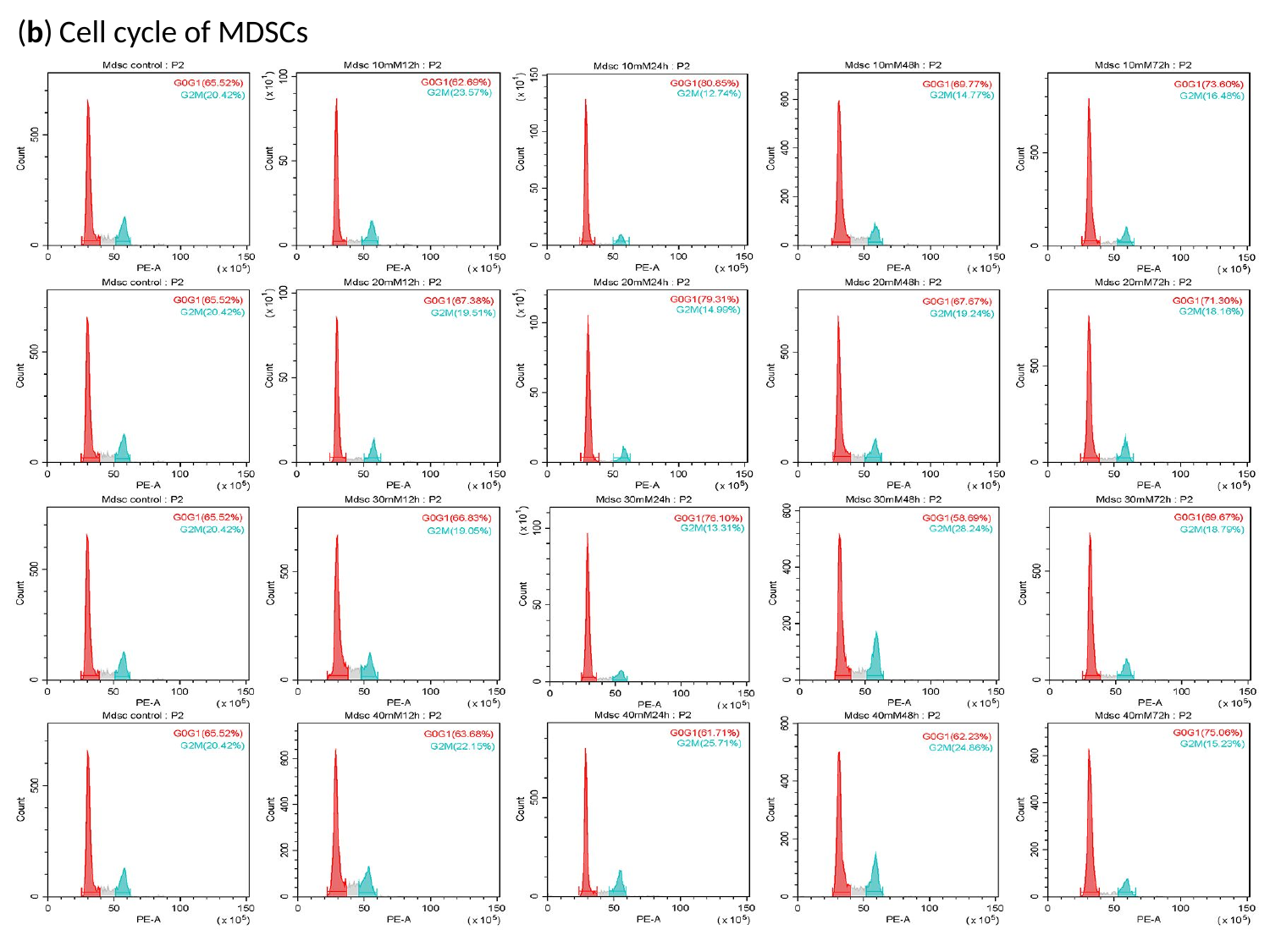

Cell cycle of MDSCs
(b)

## Slide 5
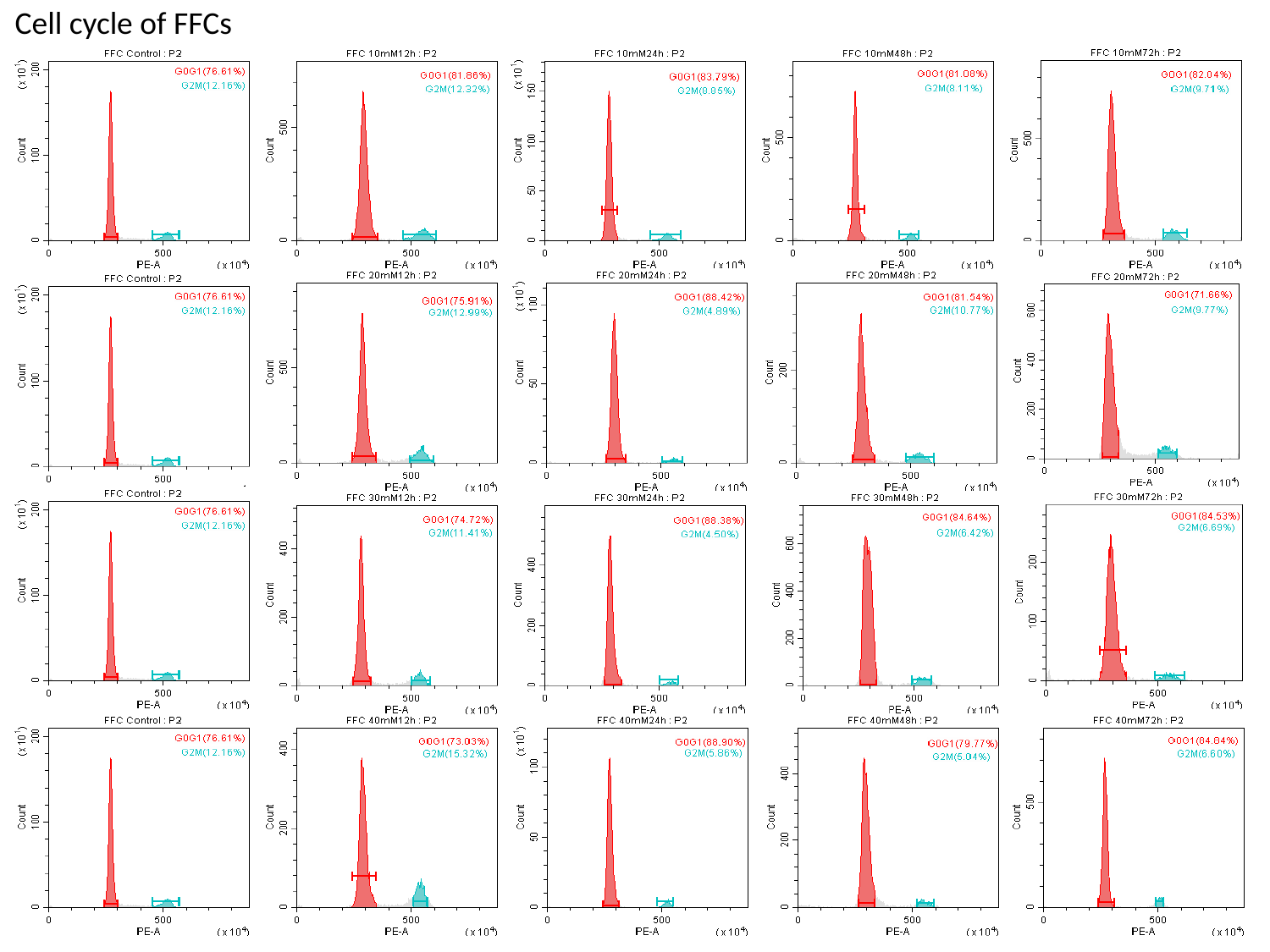

Cell cycle of FFCs

## Slide 6
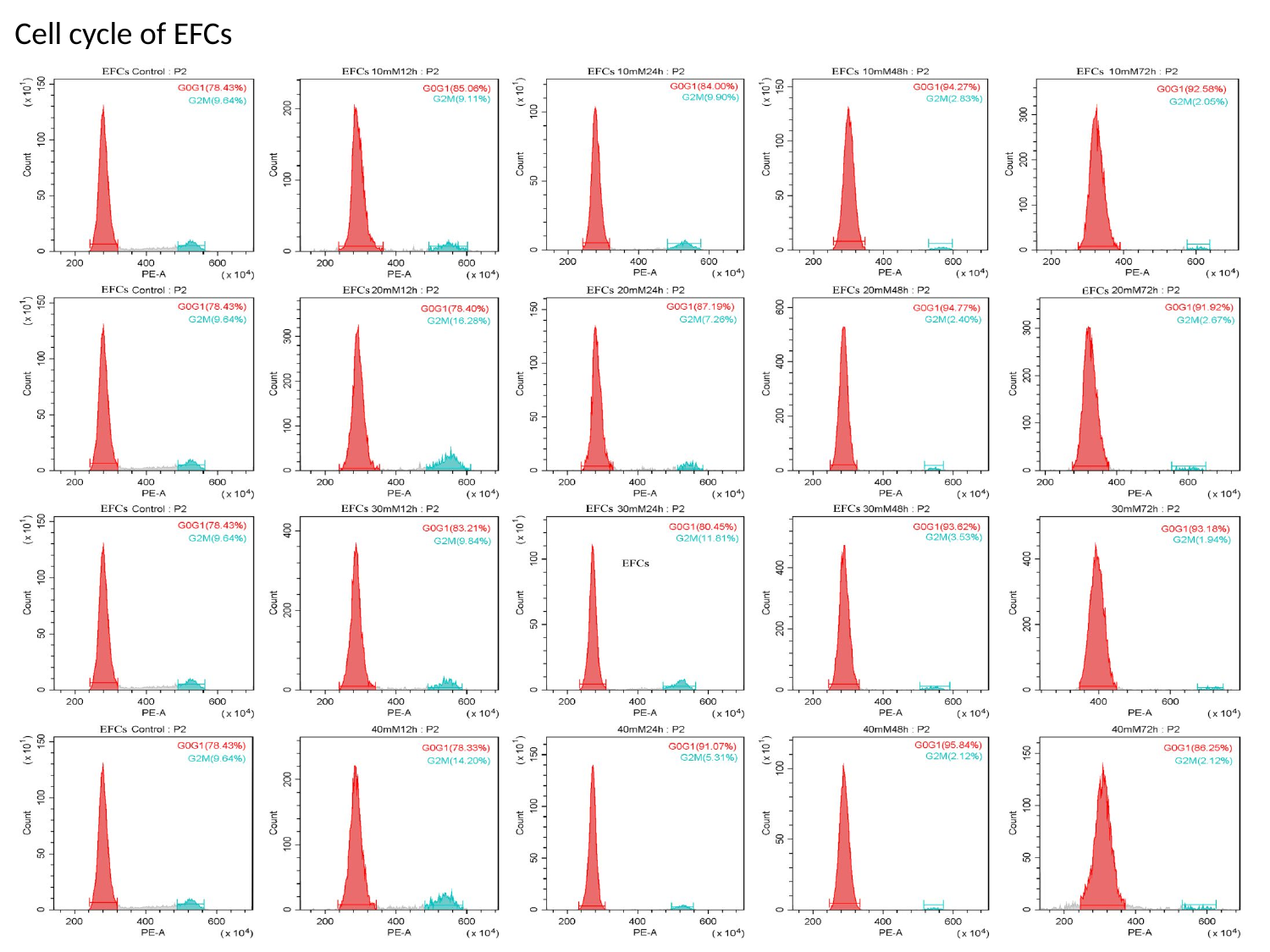

Cell cycle of EFCs

## Slide 7
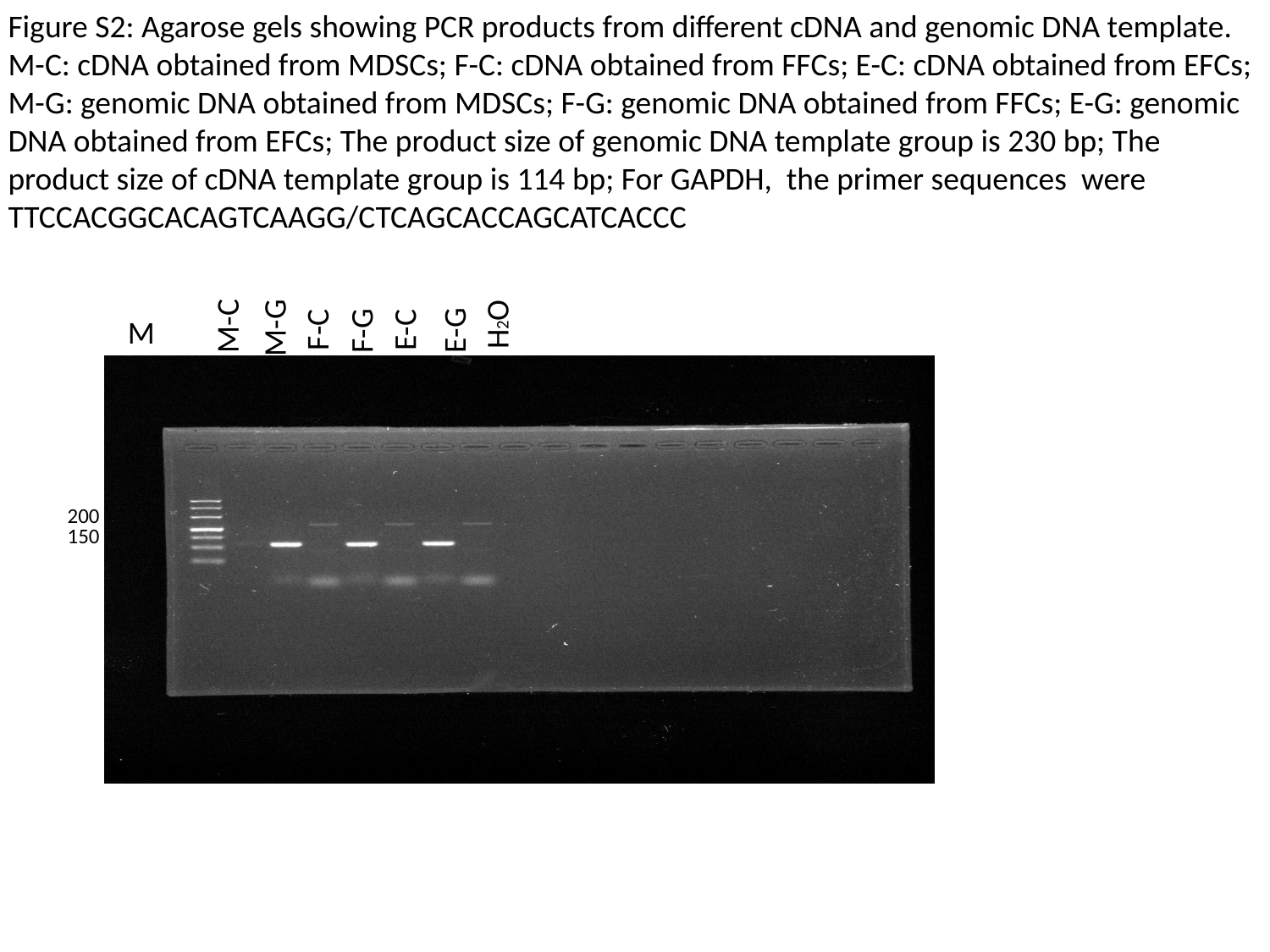

Figure S2: Agarose gels showing PCR products from different cDNA and genomic DNA template. M-C: cDNA obtained from MDSCs; F-C: cDNA obtained from FFCs; E-C: cDNA obtained from EFCs; M-G: genomic DNA obtained from MDSCs; F-G: genomic DNA obtained from FFCs; E-G: genomic DNA obtained from EFCs; The product size of genomic DNA template group is 230 bp; The product size of cDNA template group is 114 bp; For GAPDH, the primer sequences were TTCCACGGCACAGTCAAGG/CTCAGCACCAGCATCACCC
H2O
M-C
M-G
F-C
E-C
E-G
F-G
M
200
150

## Slide 8
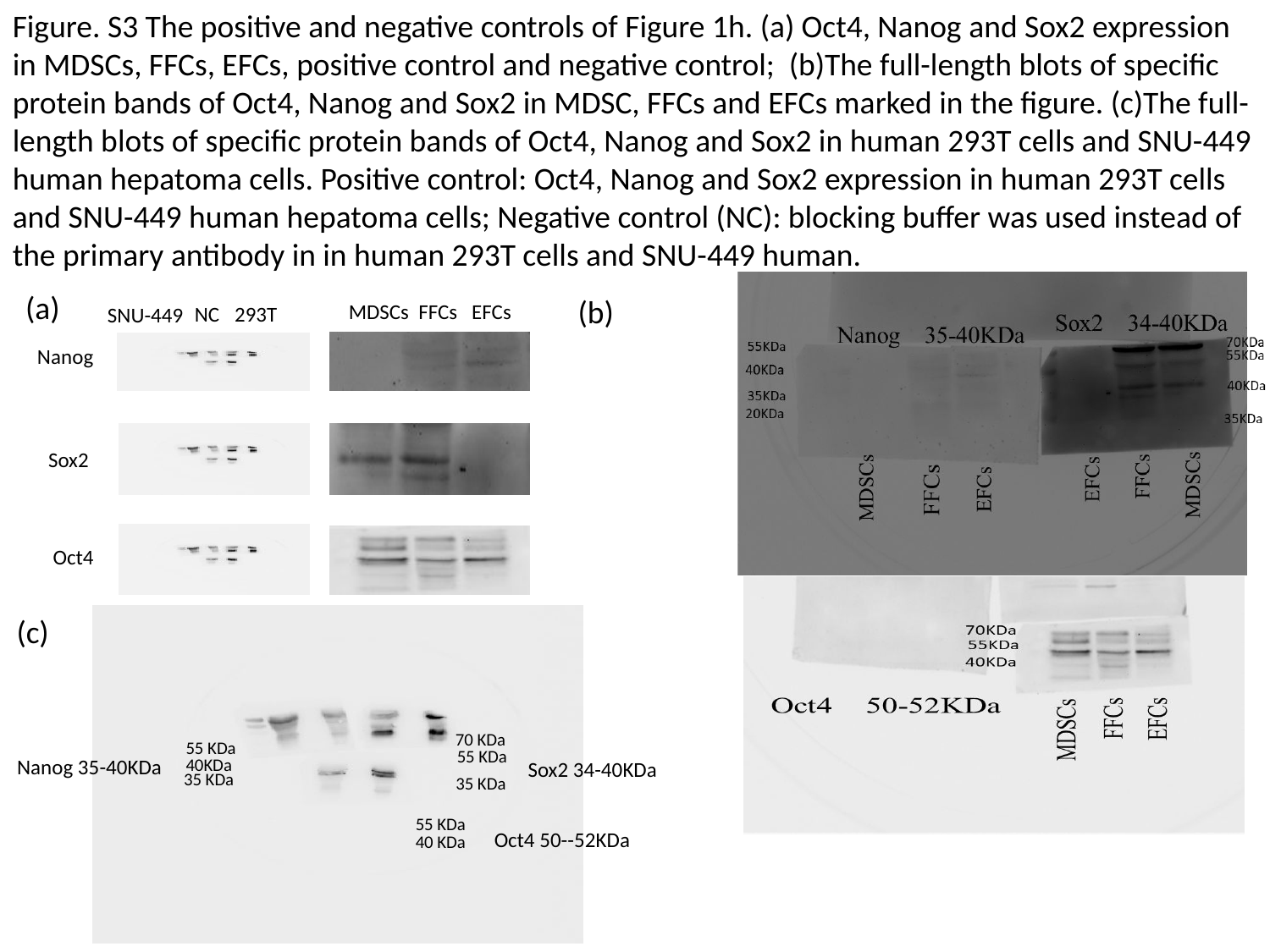

Figure. S3 The positive and negative controls of Figure 1h. (a) Oct4, Nanog and Sox2 expression in MDSCs, FFCs, EFCs, positive control and negative control; (b)The full-length blots of specific protein bands of Oct4, Nanog and Sox2 in MDSC, FFCs and EFCs marked in the figure. (c)The full-length blots of specific protein bands of Oct4, Nanog and Sox2 in human 293T cells and SNU-449 human hepatoma cells. Positive control: Oct4, Nanog and Sox2 expression in human 293T cells and SNU-449 human hepatoma cells; Negative control (NC): blocking buffer was used instead of the primary antibody in in human 293T cells and SNU-449 human.
(a)
(b)
MDSCs FFCs EFCs
NC
293T
SNU-449
Nanog
Sox2
Oct4
(c)
70 KDa
55 KDa
55 KDa
Nanog 35-40KDa
40KDa
Sox2 34-40KDa
35 KDa
35 KDa
55 KDa
Oct4 50--52KDa
40 KDa

## Slide 9
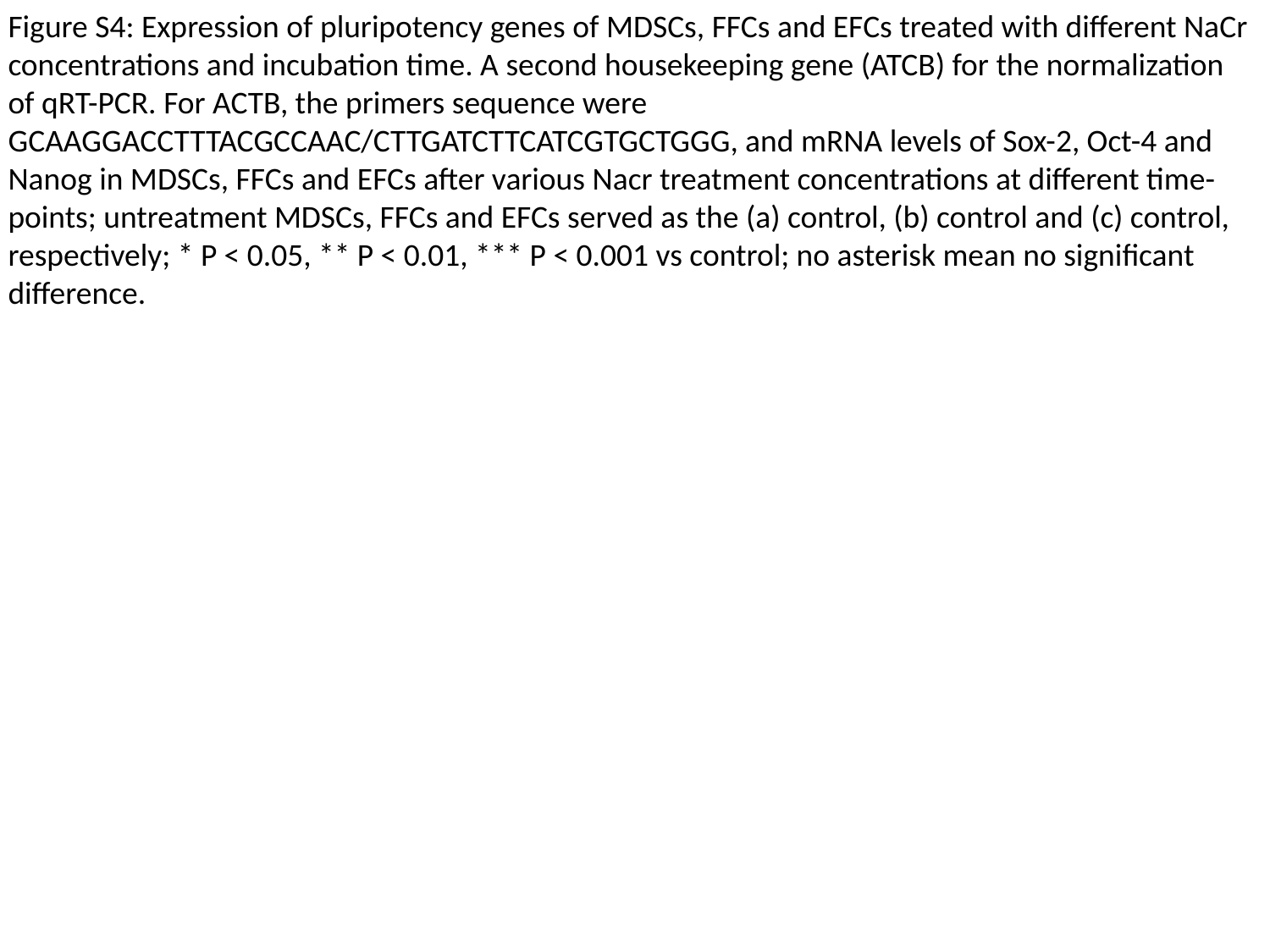

Figure S4: Expression of pluripotency genes of MDSCs, FFCs and EFCs treated with different NaCr concentrations and incubation time. A second housekeeping gene (ATCB) for the normalization of qRT-PCR. For ACTB, the primers sequence were GCAAGGACCTTTACGCCAAC/CTTGATCTTCATCGTGCTGGG, and mRNA levels of Sox-2, Oct-4 and Nanog in MDSCs, FFCs and EFCs after various Nacr treatment concentrations at different time-points; untreatment MDSCs, FFCs and EFCs served as the (a) control, (b) control and (c) control, respectively; * P < 0.05, ** P < 0.01, *** P < 0.001 vs control; no asterisk mean no significant difference.

## Slide 10
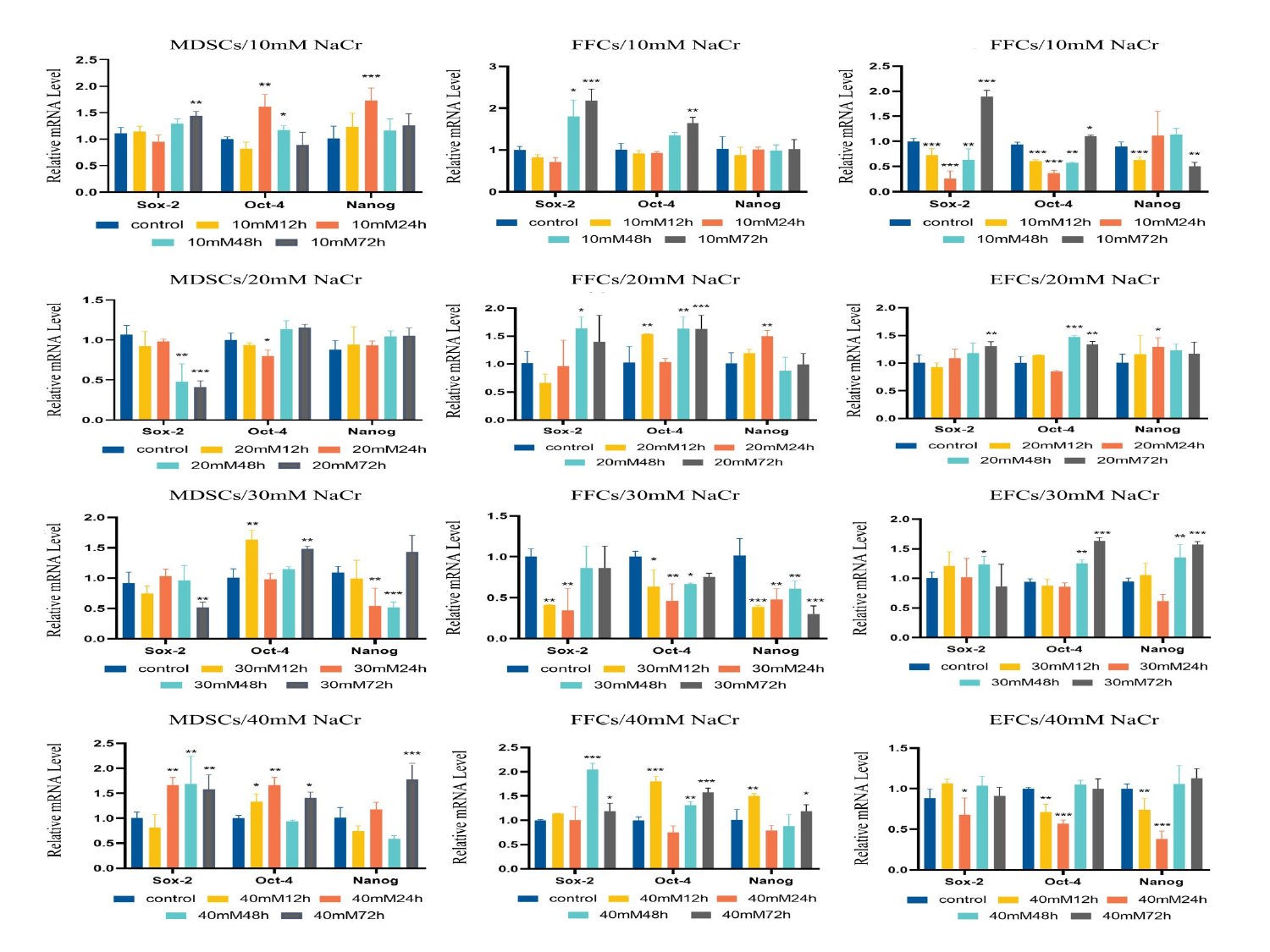

Supplement: Supplementary file 1 [file animals-12-02848-s001.zip › Supplementary Figure.pptx]
